# 723. *Cryptosporidium* Detection in Preserved Stool Specimens: A Comparison Study of EIA, DFA, and Direct Microscopic Method

**DOI:** 10.1093/ofid/ofab466.920

**Published:** 2021-12-04

**Authors:** Jian R Bao, Ronald N Master, Robert S Jones, Richard B Clark, Kileen L Shier

**Affiliations:** 1 Quest Diagnostics Nichols Institute, Chantilly, Virginia; 2 Nichols Institute, Quest Diagnostics, Secaucus, New York

## Abstract

**Background:**

*Cryptosporidium* is an intestinal parasite that may cause diarrhea. Laboratory diagnosis largely relies on microscopic or immunology-based antigen detection. Direct fluorescent antibody (DFA) is considered the gold standard. Enzyme immunoassay (EIA) is an attractive alternative, but direct comparison studies for the performance together with the impact from different specimen preservation media are limiting.

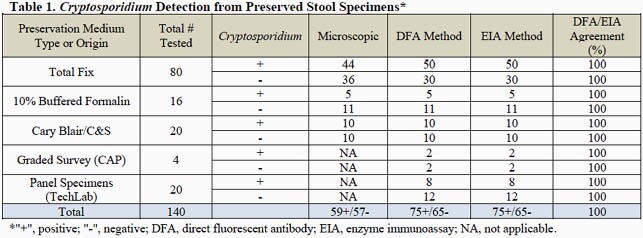

**Methods:**

We compared these three methods for the detection of *Cryptosporidium* oocysts (direct microscopic) or antigen (DFA or EIA) from stool samples preserved in either 10% buffered formalin, Cary-Blair/C&S, or Total Fix (MCC, Torrance, CA). The DFA from Meridian Bioscience (Cincinnati, OH) and the EIA using *CRYPTOSPORIDIUM II* (TechLab^®^, Blacksburg, VA) were performed according to the manufacturer’s instructions. The direct microscopic method was performed according to laboratory protocols, including direct wet mount, modified acid-fast stain, or permanent trichrome stain.

**Results:**

A total of 140 samples, including 116 clinical specimens, 20 validation panel samples and 4 proficiency survey specimens, were examined (Table 1). The DFA and EIA methods produced 100% concordant results using all three preservatives, while the microscopic method had decreased sensitivity. All microscopic positives remained positive for both the DFA and EIA. Cross-reactivity from other parasites, such as *Giardia*, of the two immunoassays was not observed.

**Conclusion:**

While the two immunological methods both outperformed the microscopic method, the EIA has the advantages of being objective, simple to perform, has less hands-on time, and thus makes it an attractive option for high throughput *Cryptosporidium* detection.

**Disclosures:**

**Kileen L. Shier, PhD, D(ABMM), MLS(ASCP)CM**, Quest Diagnostics (Employee)

